# Abundance and population growth estimates for bare‐nosed wombats

**DOI:** 10.1002/ece3.10465

**Published:** 2023-09-04

**Authors:** Wiebke Knoblauch, Scott Carver, Michael M. Driessen, Rosemary Gales, Shane A. Richards

**Affiliations:** ^1^ Department of Biological Sciences University of Tasmania Hobart Tasmania Australia; ^2^ Department of Natural Resources and Environment Tasmania Hobart Tasmania Australia; ^3^ School of Natural Sciences University of Tasmania Hobart Tasmania Australia

**Keywords:** bare‐nosed wombat, distance sampling, geographically widespread species, population estimate, spotlight survey

## Abstract

Wildlife managers often rely on population estimates, but estimates can be challenging to obtain for geographically widespread species. Spotlight surveys provide abundance data for many species and, when conducted over wide spatial scales, have potential to provide population estimates of geographically widespread species. The bare‐nosed wombat (*Vombatus ursinus*) has a broad geographical range and is subject to spotlight surveys. We used 19 years (2002–2020) of annual spotlight surveys to provide the first estimates of population abundance for two of the three extant bare‐nosed wombat subspecies: *V. u. ursinus* on Flinders Island; and *V. u. tasmaniensis* on the Tasmanian mainland. Using distance sampling methods, we estimated annual rates of change and 2020 population sizes for both subspecies. Tasmanian mainland surveys included habitat data, which allowed us to also look for evidence of habitat associations for *V. u. tasmaniensis*. The average wombat density estimate was higher on Flinders Island (0.42 ha^−1^, 95% CI = 0.25–0.79) than on the Tasmanian mainland (0.11 ha^−1^, CI = 0.07–0.19) and both wombat subspecies increased over the 19‐year survey period with an estimated annual growth rate of 2.90% (CI = −1.7 to 7.3) on Flinders Island and 1.20% (CI = −1.1 to 2.9) on mainland Tasmania. Habitat associations for *V. u. tasmaniensis* were weak, possibly owing to survey design; however, we detected regional variation in density for this subspecies. We estimated the population size of *V. u. ursinus* to be 71,826 (CI = 43,913–136,761) on Flinders Island, which when combined with a previously published estimate of 2599 (CI = 2254–2858) from Maria Island, where the subspecies was introduced, provides a total population estimate. We also estimated 840,665 (CI = 531,104–1,201,547) *V. u. tasmaniensis* on mainland Tasmania. These estimates may be conservative, owing to individual heterogeneity in when wombats emerge from burrows. Although these two subspecies are not currently threatened, our population estimates provide an important reference when assessing their population status in the future, and demonstrate how spotlight surveys can be valuable to inform management of geographically widespread species.

## Graphical abstract

Population estimates are important for wildlife management and conservation efforts but often difficult to obtain; especially for geographically widespread species due to high labour and logistic costs. We address this problem by focussing on a commonly applied survey method (spotlight survey) for a geographically widespread species (bare‐nosed wombat), including easily obtainable data (distance data).

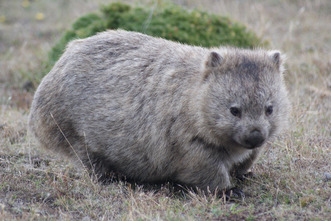

## 1 INTRODUCTION

Population estimation of geographically widespread species poses important problems for wildlife management owing to the challenges of undertaking representative surveys across large spatial scales (Houlahan et al., 2000; Witmer, 2005), and high logistic and labour costs associated with undertaking spatially dispersed study designs (Conrad et al., 2004; Pollock et al., 2002; Senyatso et al., 2013; Thakur et al., 2018). Widespread species are often perceived as relatively immune to threatening processes, however, this is not always true (Gaston & Fuller, 2007; Thakur et al., 2018; Woinarski et al., 2015). At worst, inadequate or spatially restricted population assessment can fail to detect population declines and extirpation events in areas across a species' range (Ramp & Ben‐Ami, 2006; Roger et al., 2007; Senyatso et al., 2013), and the need for intervention may only become apparent when interventions are no longer effective (Woinarski et al., 2015). The application of survey methods that are feasible over large spatial scales, and repeated over time, represents significant value for species conservation and management (Carver et al., 2021; Jones, 2011).

Repeated monitoring methods that produce indices of abundance (e.g. indirect indices based upon animal signs, such as: scats, tracks and burrows, and direct signs, such as: camera trapping, satellite imagery and spotlight surveys) are generally more feasible for widespread species when compared to more intensive methods (e.g. capture–mark–recapture surveys) and are valuable as they allow an assessment of relative population development. Understanding how indices translate to abundance is highly desirable in wildlife conservation and management (Conroy & Nichols, 1996; Witmer, 2005). For example, the International Union for Conservation of Nature often relies on population estimates for determining species conservation status (IUCN, 2012). Fortunately, many direct observation indices can incorporate distance sampling methods, which can provide both estimates of abundance and population change when surveys are repeated over time. Such abundance estimates can assist efforts to assess and manage geographically widespread wildlife species (Hopkins & Kennedy, 2004).

The bare‐nosed or ‘common’ wombat (*Vombatus ursinus*; Shaw 1800) is one of Australia's iconic marsupials and occupies a broad geographical range across south‐eastern Australia. Bare‐nosed wombats comprise three subspecies: the Australian mainland *Vombatus ursinus hirsutus* (Perry 1810) occurs discontinuously from south‐eastern Queensland to south‐eastern South Australia; the Bass Strait ‘Flinders Island wombat’ *V. u. ursinus* (Shaw 1800) (AMTC, 2022) on Flinders and Maria islands; and the Tasmanian mainland *V. u. tasmaniensis* (Spender and Kershaw, 1919) (Jackson & Groves, 2015; Martin et al., 2019). Across their range, bare‐nosed wombats are found in a broad range of habitats from sea level to alpine elevations (Driessen, Dewar, Carver, Lawrence, & Gales, 2022; McIlroy, 2008). Bare‐nosed wombats occupy an important ecological role as ‘ecosystem engineers’. Their burrowing behaviour helps turn over soil which assists with water infiltration and nutrient cycling (Borchard et al., 2008; Evans, 2008; Old et al., 2018) and wombat burrows also provide habitat for a range of other species (e.g. wallabies, possums and Tasmanian devils; Kinlaw, 1999; Simpson et al., 2016; Old et al., 2018). Currently, bare‐nosed wombats are not listed as threatened under any Australian species legislation and are classified as ‘least concern’ with a ‘stable population’ on the IUCN Red List due to perceived ‘commonness’ and wide geographical distribution (Driessen, Dewar, Carver, Lawrence, & Gales, 2022; Taggart et al., 2016). Despite their broad geographical range, all three wombat subspecies have experienced historical range declines associated with European settlement (Cooke, 1998; Triggs, 2009). In addition to habitat alteration and destruction, wombats have suffered persecution owing to their actual or perceived damage to agricultural land or infrastructure (Triggs, 2009). Other threats include introduced predators, namely wild dogs and foxes (Triggs, 2009), vehicle collisions (Driessen, Dewar, Carver, Lawrence, & Gales, 2022; Nguyen et al., 2019; Ramp et al., 2005; Roger et al., 2007) and sarcoptic mange disease (Driessen, Dewar, Carver, & Gales, 2022; Martin et al., 2018; Stannard et al., 2021). Research is needed to understand the population status of this geographically widespread species.

While the range of the bare‐nosed wombat is broadly understood, the size of the population within their existing range remains a knowledge gap. Various studies have used indices of abundance to monitor local population trends or identify habitat usage; for example, wombat burrows have been shown to be associated with gullies, waterways, shrubby habitats and proximity to forest cover (McIlroy, 1973; Roger et al., 2007; Triggs, 2009). Burrow counts by Catling et al. (2000) suggested a preference of bare‐nosed wombats for open forests with an open grassy understorey and low shrub cover. Similarly, Taylor (1993) observed numerous burrows in sown pasture areas, all of which were located within patches of shrub. Even though there is a small number of spatially restricted studies on bare‐nosed wombat population densities in specific habitats (Buchan & Goldney, 1998; Evans, 2008; Mallett & Cooke, 1986; McIlroy, 1977; Skerratt et al., 2004; Taylor, 1993), the extent to which these studies are broadly representative is unknown.

Here, we estimate population sizes and recent population trends for two of the three extant bare‐nosed wombat subspecies: *V. u. ursinus* on Flinders Island and *V. u. tasmaniensis* on the Tasmanian mainland. We used 19 years of spotlight survey data collected by the Tasmanian State Government (Department of Natural Resources and Environment [NRE]), which encompasses most of the range of these two subspecies. Habitat data collected during the Tasmanian mainland surveys also allowed us to look for evidence of habitat associations, and helped with assessing uncertainty associated with our population estimates.

## 2 METHODS

### 2.1 Study area

**FIGURE 1:**
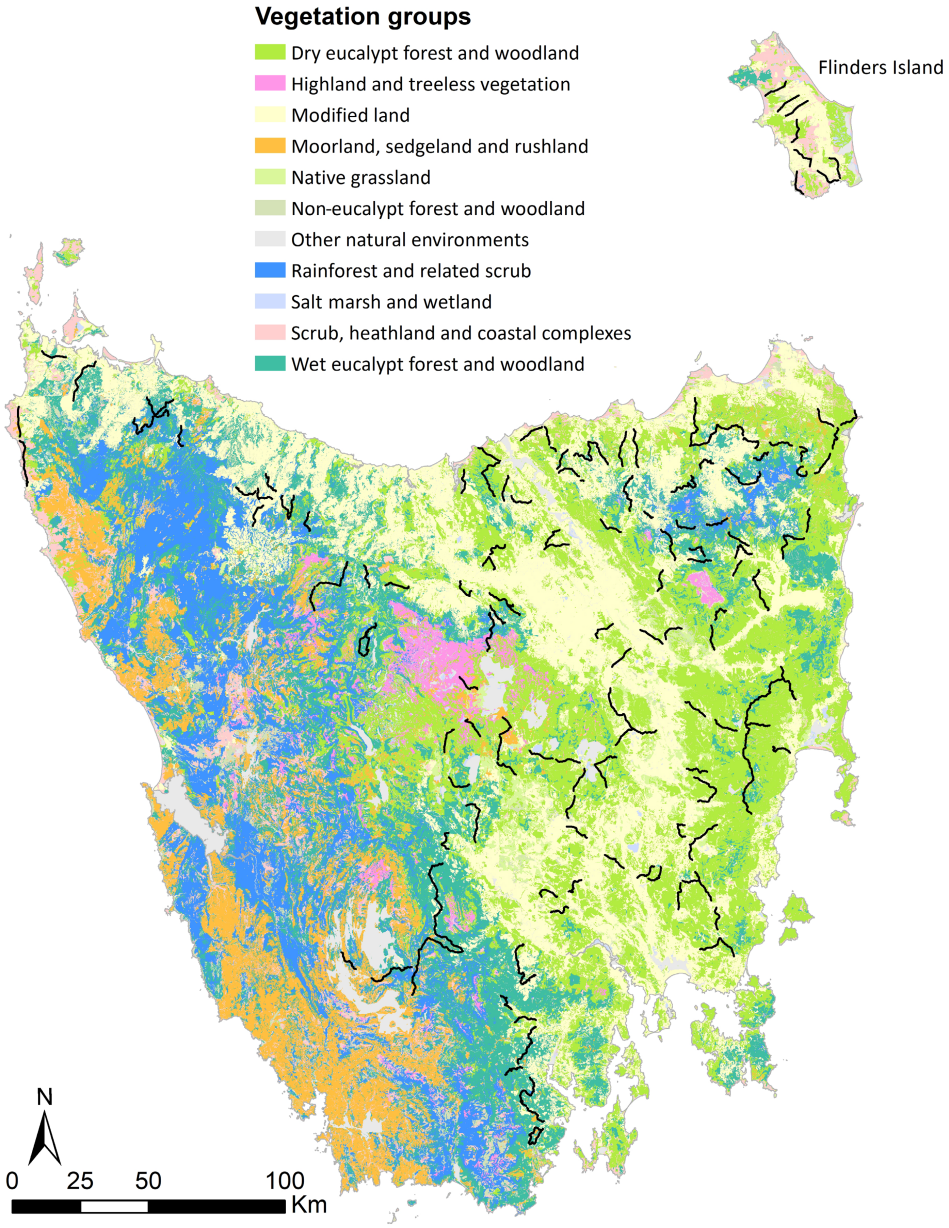
Tasmania, Australia, with broad groupings of vegetation communities as defined by Kitchener and Harris (2013). Black lines are 180 10‐km spotlight transects (172 on the Tasmanian mainland, 8 on Flinders Island), used for annually conducted wildlife surveys by the Department of Natural Resources and Environment (NRE), Tasmania.

The island state of Tasmania is approximately 68,102 km^2^ in size (Australian Bureau of Statistics, 2008) and has a mostly temperate maritime climate. Maximum temperatures range between 18°C and 23°C in summer and between 9°C and 14°C in winter, but with elevated regions (>500 m) experiencing temperatures about 5°C lower than that (Bureau of Meteorology, 2021; Commonwealth of Australia, 1999). The mid‐latitude westerlies, the prevailing westerly airstream, directly affects Tasmania's climate and forms a gradient: the West Coast and elevated regions are cool, wet and cloudy while the East Coast and lower regions are generally mild, dry and sunny. Rainfall also varies, with an average of <600 mm in Tasmania's Midlands, >3500 mm in parts of the west where it is relatively mountainous, and around 800 mm along the North West Coast (Commonwealth of Australia, 1999). Western Tasmania is dominated by moorland, alpine heath, rainforest, sclerophyll forest and scrub land while coastal heath, dry sclerophyll forest and woodland dominate the east (Figure 1; Department of Natural Resources and Environment, 2020; Roe & Scott, 2021). Due to its mountainous nature and lower soil fertility in the west and south, most agriculture is restricted to the east and the north of the island (Figure 1). More than 50% of Tasmania's land area is reserved for conservation of which the major part is in the island's west (Department of Natural Resources and Environment, 2021a).

Flinders Island has an area of 1360 km^2^ (Department of Natural Resources and Environment, 2020) and is located north of the Tasmanian mainland (Figure 1). The annual average rainfall is 741 mm, being highest in winter and lowest in summer (Bureau of Meteorology, 2021). Mean maximum and minimum temperatures range from 23°C and 14°C in February to 13°C and 6°C in July (Bureau of Meteorology, 2021). For the most part, Flinders Island is low‐lying, but with a granite mountain range running along the length of the island, rising to Strzelecki Peaks (778 m) in the south (Ladd et al., 1992). Lower areas are used for pasture (mainly for sheep) while the remaining areas are dominated by heathlands, shrublands, sand dunes and lagoons (Figure 1; Department of Natural Resources and Environment, 2020; Natural and Cultural Heritage Division, 2014).

### 2.2 Spotlight surveys

We used wombat count and distance data collected annually as part of the Tasmanian mammal spotlight survey conducted by the Tasmanian NRE (NRE Tas). This survey was established to monitor population trends in species subjected to regular culling such as the Bennett's Wallaby (*Notamacropus rufogriseus*; Desmarest, 1817), the Tasmanian pademelon (*Thylogale billardierii*; Desmarest 1822) and the Brushtail possum (*Trichosurus vulpecula*; Kerr, 1792). However, other mammal species were also recorded during the surveys (Driessen & Hocking, 1992).

Between November and February (austral summer) each year from 2002 to 2020, trained departmental officers conducted spotlight counts from a vehicle along 172 transects located throughout the north‐west and north‐east as well as central and south‐eastern parts of mainland Tasmania (Figure 1). Further, eight transects were surveyed on Flinders Island (Figure 1) in the same time period. Even though wombats occur in the south‐west and much of the west of Tasmania, these regions do not have transects due to limited vehicular access and a lack of regular culling (Carver et al., 2021). All 180 transect lines comprise a 10‐km section of road and adjacent land.

Two people conducted each survey; the driver operated a 100‐watt handheld spotlight while travelling at ≤20 km/h and counted wildlife on both sides of the road, relaying their observations to a passenger for recording (Department of Natural Resources and Environment, 2021b). During a night, observers generally surveyed five to seven transects in a set order, starting half an hour after sunset. Even though surveys are conducted under various weather conditions, periods of heavy rain, fog or high winds were generally avoided. For each counted wombat, perpendicular distance from transect was recorded, using six categories: 0–5, 5–10, 10–20, 20–40, 40–60 and 60–100 m. Distances were measured using a laser rangefinder (*Bushnell Yardage Pro 600 Compact Laser Rangefinder*; Robbie Gaffney, personal communication, October 2021). While the number of wombats on each transect was recorded, the specific co‐ordinates of each observation was not available.

### 2.3 Predictor variables and data aggregation

**FIGURE 2:**
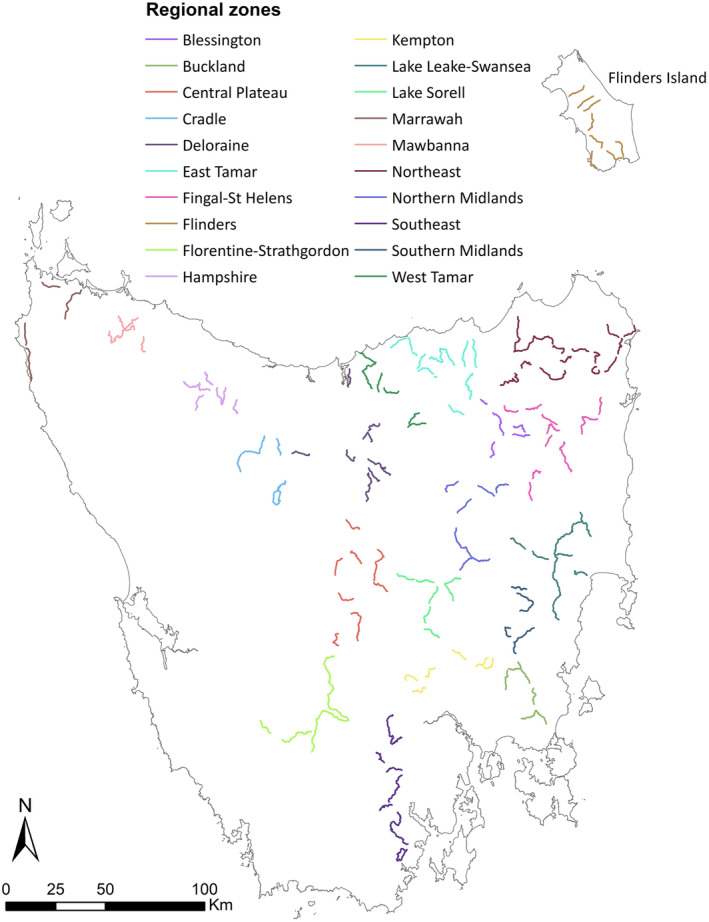
Tasmania, Australia, with 180 10‐km spotlight transects (172 on the Tasmanian mainland, 8 on Flinders Island). On the Tasmanian mainland, bare‐nosed wombat (*Vombatus ursinus tasmaniensis*) counts and distance data were recorded from transects spanning 19 regional zones (denoted by transect colour). Flinders Island was treated as single zone comprising the Flinders Island wombat (*Vombatus ursinus ursinus*).

Transect counts of wombats were analysed at two levels: (1) broad vegetation groups (Figure 1) as defined by Kitchener and Harris (2013) and used in the TASVEG 4.0 dataset (Department of Natural Resources and Environment, 2020); and (2) regional zones (Figure 2) as described in Carver et al. (2021), which are aligned with geographical locations but not defined by specific spatial boundaries. Using the ArcMap application within ArcGIS v.10.6. (Environmental Systems Research Institute ESRI, 2018) and a polyline layer comprising all transect routes, a buffer of 100 m was created around the transects to reflect the spatial scope in which wombats were mostly seen from the transect. By visual assessment, we subdivided each buffer zone into proportions of present vegetation group and assigned those to the respective transect line because vegetation associations of wombats are known to occur (Burgess et al., 2023; Ringwaldt et al., 2023). This was done only for the Tasmanian mainland due to the small sample size of eight transects on Flinders Island, as well as their predominant placement in agricultural land (Figure 1; Table A1). Regional analysis was based on spatial clustering of transects and local elevation, as described by Carver et al. (2021). For the Tasmanian mainland, three extra regional zones were defined (Florentine‐Strathgordon, Kempton and Southeast which integrates the former Geeveston zone), as our study included 40 additional transects to those described in Carver et al. (2021), while Flinders Island was a separate zone (Figure 2).

### 2.4 Analysis

Statistical analyses were conducted using the program R v4.0.2 (R Development Core Team, 2020). Packages used were: tidyverse (Wickham et al., 2019), readxl (Wickham & Bryan, 2019), rstan (Stan Development Team, 2020), bayesplot (Gabry & Mahr, 2021), cowplot (Wilke, 2020) and expint (Goulet, 2016).

### 2.5 Estimating wombat density

Wombat densities of individuals per ha (hereafter ha^−1^) were estimated by developing and fitting models to the wombat observation data, taking into account that the counts were obtained via distance sampling. A key concept of all distance sampling methods is the detection function *g(x)* (Buckland et al., 2001), that is the probability of detecting an animal, given that it is at a distance *x* from the transect line. The detection probability usually declines with increasing distance from the transect and the shape of this decline, namely the detection function, can be used to estimate the proportion of animals missed during the survey (Buckland et al., 2001; Schmidt et al., 2012). Thus, distances recorded for each wombat sighting were used to estimate a detection function (Buckland et al., 2015).

**FIGURE 3:**
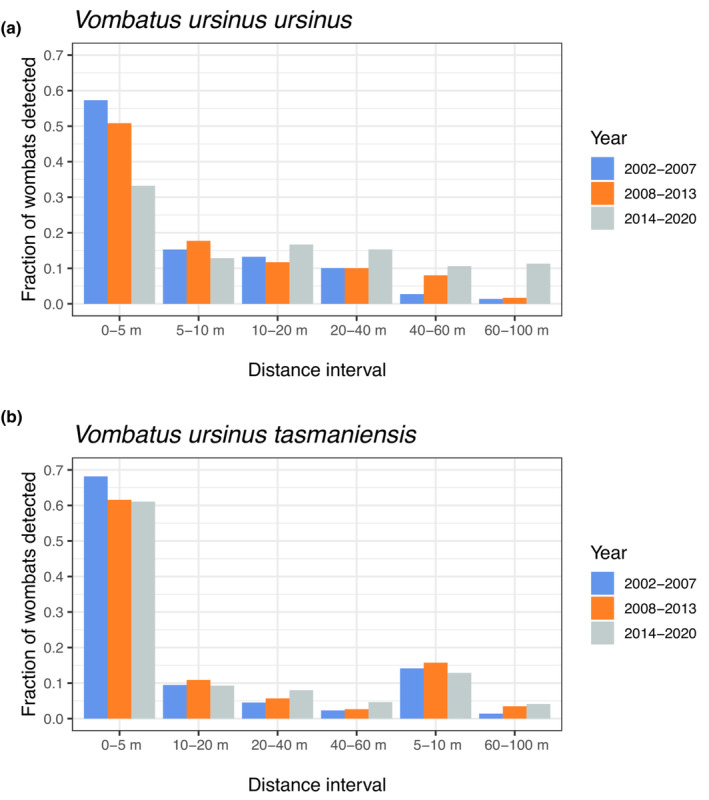
Proportions of the number of wombats detected within distance intervals during spotlight surveys from 2002 to 2020 on (a) Flinders Island (subspecies *Vombatus ursinus ursinus*) and (b) the Tasmanian mainland (subspecies *V. u. tasmaniensis*) in Australia.

Due to clear differences in observability, analyses were performed separately for the *V. u. ursinus* population on Flinders Island and the *V. u. tasmaniensis* population on the Tasmanian mainland. Specifically, the proportion of wombats detected at greater distances increased over time on Flinders Island (Figure 3a) but not on the Tasmanian mainland (Figure 3b), and detectability declined initially much more rapidly on the Tasmanian mainland (Figure 3).

#### 2.5.1 Flinders Island analysis

Figure 3a indicates a sharp initial decline in wombat detection with distance on Flinders Island, which can be described by a detection function that combines two negative exponential curves:

$$g\left(x\right)={\mathrm{qe}}^{-\frac{x}{\;\mu_{1}}}+\left(1-q\right)e^{-\frac{x}{\mu_{2}}}$$

where *x* is the perpendicular distance from the road, and *q*, *μ*_1_ and *μ*_2_ are positive constants to be determined from the data. *μ*_1_ and *μ*_2_ describe two scales of detectability; the probability of detecting a wombat at distance *μ* is 1/e = 0.37, and *q* is the weighting associated with the curve characterised by *μ*_1_.

To reflect a temporal shift in detectability that is consistent with Figure 3a, the formulation was modified by:

$$g\left(x,t\right)=q\left(t\right)e^{-\frac{x}{\mu_{1}}}+\left(1-q\left(t\right)\right)e^{-\frac{x}{\mu_{2}}}$$

where *t* is year and *q*(*t*) is described by the logistic equation:

$$\text{logit}\;q\left(t\right)=\alpha_{0}+\alpha_{1}\left(t-T\right),$$

and *T* is a reference year, which was set to 2020.

The probability of an animal being observed within distance band *i*, bounded by distances *x*_*i*_ and *x*_*i*–1_, in year *t* is:

$$p_{i,t}=\frac{1}{x_{i}-x_{i-1}}\;\int_{x=x_{i-1}}^{x_{i}}g\left(z,t\right)\;\mathrm{dz},$$

which is equivalent to:

$$p_{i,t}=\frac{1}{x_{i}-x_{i-1}}\;\left(q\left(t\right)\mu_{1}\left(e^{-\frac{x_{0}}{\mu_{1}}}-e^{-\frac{x_{1}}{\mu_{1}}}\right)+\left(1-q\left(t\right)\right)\mu_{2}\left(e^{-\frac{x_{0}}{\mu_{2}}}-e^{-\frac{x_{1}}{\mu_{2}}}\right)\right).$$

The mean density of wombats along transect j during year t is $\lambda_{j,t}$. If wombats are spotted on both sides of the transect, then the expected number of animals spotted along route j during year t, within distance category *i*, is:

$${\bar{n}}_{i,j,t}=p_{i,t}2\left(x_{i}-x_{i-1}\right)l_{j}\lambda_{j,t}$$

where $l_{j}$ is the length of transect j. The variation about this expectation was presumed to be consistent with the negative binomial distribution with a constant variance term, Φ; if the mean is $\bar{n}$, then the variance is $\left(1+\Phi \right)\bar{n}$ (Richards, 2008).

In addition, we assumed that, on average, wombat abundance has been increasing at rate r over time for all transects and mean wombat density in 2020 is λ. Relative wombat abundance may differ between transects due to unaccounted for transect variation, and there may be wide‐scale inter‐annual variation in wombat numbers (i.e. relatively high and low annual numbers). These patterns can be described by setting:

$${\bar{\lambda }}_{j,t}=\lambda \exp\;\left(r\left(t-T\right)+\delta_{j}+\epsilon_{t}\right),$$

where $\delta_{j}$ and $\epsilon_{t}$ are drawn from normal distributions with mean 0 and standard deviations $\sigma_{R}$ and $\sigma_{Y}$, respectively (i.e. they describe random effects).

#### 2.5.2 Tasmanian mainland analysis

Figure 3b suggests a simpler detection function can describe wombat sightings for the Tasmanian mainland surveys; namely,

$$g\left(x\right)=\kappa +\left(1-\kappa \right)e^{-\alpha_{l}x}$$

where κ is the minimum probability of observing a wombat at any distance, and $\alpha_{l}$ is the decline in detection associated with transect l. Unlike Flinders Island, there was no evidence that the detection curve changed over the years surveyed.

For the Tasmanian mainland, where habitat and region information were available, the expected density of wombats in transect j in year t was described by:

$${\bar{\lambda }}_{j,t}=\lambda \exp\;\left(r\left(t-T\right)+\delta_{j}+\epsilon_{t}+\eta_{m}+\sum_{i=1}^{n}p_{i,j}e^{\beta_{i}}\right),$$

where $\delta_{j}$ accounts for transect variation, $\epsilon_{t}$ for inter‐annual variation and $\eta_{m}$ for region variation. These parameters were assumed to be drawn from normal distributions with mean 0 and standard deviations $\sigma_{T}$, $\sigma_{Y}$ and $\sigma_{R}$, respectively. The $\beta_{i}$ describe the relative effect of habitat type i on wombat density. The proportion of transect j that is classified as being habitat i is given by $p_{i,j}$.

#### 2.5.3 Parameter estimation

Both models were coded in R using the Stan programming language (Carpenter et al., 2017), and model parameters were estimated using Bayesian distance methods (Gabry & Mahr, 2021). For all parameters, diffuse prior distributions were used such that parameter uncertainty was dominated by the data and not the prior. Markov chain Monte Carlo (MCMC) methods were used to estimate the posterior distributions of the parameters. Two chains of length 400 iterations for each parameter with a burn‐in period of 200 iterations were analysed. Convergence of the Markov chains was assessed following Gelman and Hill (2007).

### 2.6 Estimating wombat abundance

As there was little differentiation of transects among vegetation groups, or regional zones, for Flinders Island (see method section), wombat abundance was simply estimated by multiplying the 2020 density estimate with the area (ha) of the island (Table A1). For the Tasmanian mainland population, total wombat abundance ($\bar{\gamma }$) was estimated according to:

$$\bar{\gamma }=\lambda \sum_{i=1}^{n}a_{n}\exp\left(\beta_{n}\right),$$

where $a_{n}$ denotes the total area (ha, Table A2) of vegetation group n on mainland Tasmania, and λ and $\beta_{n}$ are as defined above. This calculation provided an estimate of total wombat abundance on mainland Tasmania for 2020. Because some mainland Tasmania habitat types were poorly represented on the transect surveys, we also made some simple ‘conservative estimates of total wombat abundance’ to contrast their effect on our understanding of abundance estimates (see results). Conservative estimates were achieved by subtracting the estimated wombat abundance of each under‐represented habitat type from the total mainland wombat abundance, thus assuming no wombats occurred in these habitat types.

## 3 RESULTS

**FIGURE 4:**
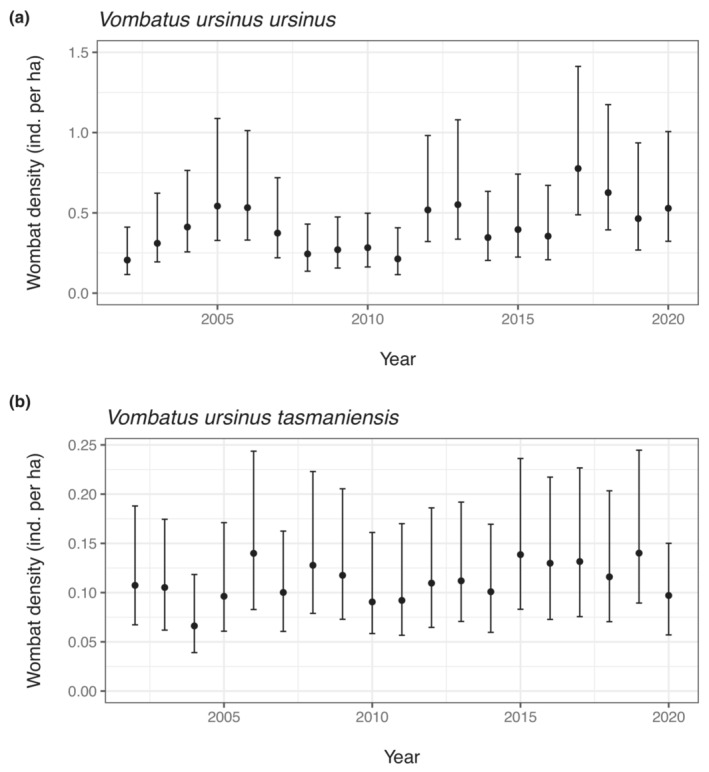
Median estimated densities for the bare‐nosed wombat populations between 2002 and 2020 on (a) Flinders Island (subspecies *Vombatus ursinus ursinus*) and (b) the Tasmanian mainland (subspecies *V. u. tasmaniensis*) in Australia. Bars indicate 95% credible intervals.

Estimates of annual wombat densities from 2002 to 2020 were higher on Flinders Island than on the Tasmanian mainland (Figure 4). The average median density estimate across all years was almost four times higher on Flinders Island (0.42 ha^−1^, 95% CI = 0.25–0.79) (Table A3) than on the Tasmanian mainland (0.11 ha^−1^, 95% CI = 0.07–0.19) (Table A4). The estimated annual growth rate was 2.4 times higher on Flinders Island (2.90%, 95% CI = −1.7–7.3) than the Tasmanian mainland (1.20%, 95% CI = −1.1–2.9).

**FIGURE 5:**
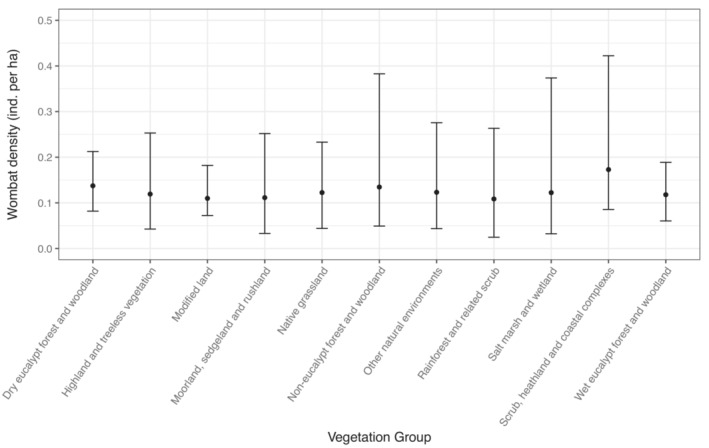
Median estimated densities for the bare‐nosed wombat on the Tasmanian mainland (*Vombatus ursinus tasmaniensis*) relating to the 11 vegetation groups as defined by Kitchener and Harris (2013). Bars indicate 95% credible intervals.

**FIGURE 6:**
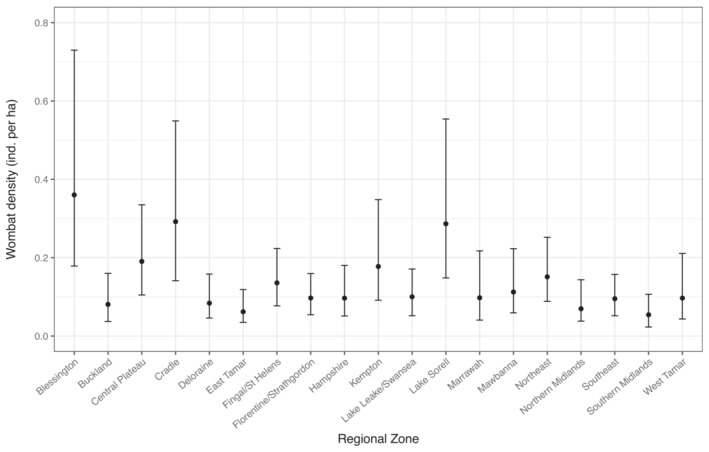
Median estimated densities for the bare‐nosed wombat on the Tasmanian mainland (*Vombatus ursinus tasmaniensis*) relating to regional zones as described in Carver et al. (2021). Three extra regional zones were added (Florentine‐Strathgordon, Kempton and Southeast which integrates the former Geeveston zone) to accommodate 40 additional transects. Bars indicate 95% credible intervals.

Estimates of density for *V. u. tasmaniensis* did not vary greatly across vegetation groups (Figure 5). In contrast, estimates of density for *V. u. tasmaniensis* varied among regions, with highest densities in Blessington, Cradle and Lake Sorell and lowest densities in the East Tamar and Southern Midlands (Figure 6).

The total abundance of *V. u. ursinus* on Flinders Island (135,897 ha) in 2020 was estimated to be 71,826 (95% CI = 43,913–136,761) while the corresponding number for *V. u. tasmaniensis* on the Tasmanian mainland (6,561,553 ha) was estimated to be 840,665 (95% CI = 531,104–1,201,547).

**FIGURE 7:**
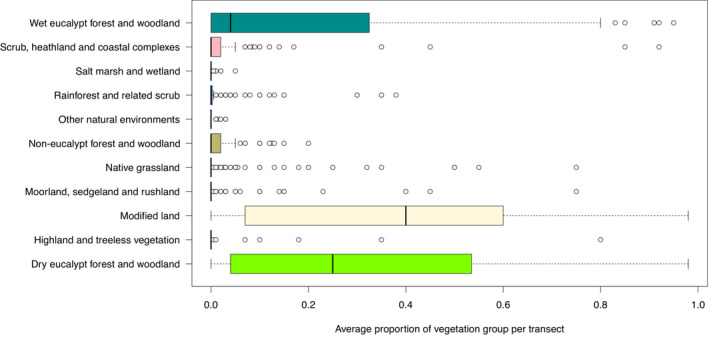
Proportion of vegetation group per spotlight survey transect (*n* = 172 10‐km transects) on mainland Tasmania, Australia (boxes = interquartile range (IQR), bars = median proportion, whiskers = variability outside of IQR, circles = outliers).

Though we were unable to determine marked variation in *V. u. tasmaniensis* densities among vegetation groups, we recognise that some habitats in Tasmania are under‐represented in the spotlight survey transects (Figures 1 and 7) and some of these may contain few wombats (Driessen, Dewar, Carver, Lawrence, & Gales, 2022; Harrison‐Day & Kirkpatrick, 2019; Kirkpatrick & Harris, 1999). Thus, we also calculated two conservative Tasmanian mainland wombat population size estimates. The first of these conservative estimates was with all habitats poorly represented in the spotlight surveys excluded (‘saltmarsh and wetland’; ‘rainforest and related scrub’; ‘other natural environments’; ‘native grassland’; ‘moorland, sedgeland and rushland’; and ‘highland and treeless vegetation’), giving an estimate of 592,748 wombats (95% CI = 340,747–1,029,441) across a land area of 4,682,666 ha. The second conservative estimate recognised that, although under‐represented in the spotlight survey transects, ‘native grassland’ and ‘moorland, sedgeland and rushland’ are a preferred habitat of wombats (Driessen, 2007; Driessen, Dewar, Carver, Lawrence, & Gales, 2022; Whinam & Hope, 2005). Thus, ‘salt marsh and wetland’; ‘rainforest and related scrub’; ‘other natural environments’; and ‘highland and treeless vegetation’ were excluded, resulting in a population size estimate of 674,094 wombats (95% CI = 365,843–120,811) across a land area of 5,400,424 ha. Notably, both conservative population estimates are still within the CI of the overall Tasmanian mainland wombat population estimate.

## 4 DISCUSSION

Being able to understand how population indices that are feasible to conduct over wide spatial scales translate into population size estimates is valuable for considering management needs of geographically widespread species. Our population estimates suggest that, on average, the number of bare‐nosed wombats on the Tasmanian mainland and Flinders Island have slowly increased since 2002, and that population sizes are in the tens of thousands for *V. u. ursinus* on Flinders Island and hundreds of thousands (possibly over one million) for *V. u. tasmaniensis* on the Tasmanian mainland. Increasing densities of wombats on Flinders Island and the Tasmanian mainland are consistent with recent studies using indices from the Tasmanian spotlight survey over longer time scales (Carver et al., 2021; Driessen, Dewar, Carver, Lawrence, & Gales, 2022). The causes of population growth of these two subspecies are likely multi‐factorial, and potentially largely reflect favourable land‐use changes (anthropogenic creation of pasture, retention of remnant vegetation where wombats burrow) and softening attitudes to wombats leading to reduced persecution (Carver et al., 2021). This study is the first published assessment of the density and abundance for two of the three bare‐nosed wombat subspecies over large spatial scales.

Previous studies in Tasmania (Taylor, 1993) and mainland Australia (Victoria, New South Wales, South Australia) have estimated wombat densities in eucalypt forests with adjacent grassland to range from 0.12 to 1.9 ha^−1^ (Buchan & Goldney, 1998; Evans, 2008; McIlroy, 1977; Skerratt et al., 2004), in predominantly pasture areas from 0.15 to 0.6 ha^−1^ (McIlroy, 1977; Taylor, 1993), between 0.28 and 0.32 ha^−1^ in woodland (Mallett & Cooke, 1986), and up to 0.23 ha^−1^ in a pine plantation (McIlroy, 1977). In contrast, our study was less able to distinguish variation in wombat densities across different land‐use types, which likely reflects the lack of precise co‐ordinate information for each individual wombat observation (observations were at the transect level). Our transects were 10 km in length and traversed multiple vegetation groups. Thus, it was not feasible to specify the specific habitat in which each wombat was observed and this lack of resolution resulted in relatively similar density estimates across vegetation groups. We suspect that densities of some habitats may have been underestimated (e.g. pastoral land) and others overestimated (e.g. salt marsh and wetland vegetation), based on known vegetation associations for bare‐nosed wombats (Burgess et al., 2023; Evans, 2008; Evans et al., 2006; McIlroy, 1973; Ringwaldt et al., 2023; Triggs, 2009). In addition, given that all transects were adjacent to roads, it is inevitable that a number of occupied habitats were under‐represented (e.g. moorland), and it is our expectation that less utilised habitat is under‐represented. Consideration towards inclusion of co‐ordinate information in these ongoing transect surveys would be valuable to improve future understanding of wombat and other faunal vegetation relationships in Tasmania. In the meantime, two recent publications provide valuable insights related to occupancy across the state (Ringwaldt et al., 2023) and abundance at a smaller spatial scale (Burgess et al., 2023).

Despite not identifying local habitat as a strong predictor of wombat density, we were able to detect regional variation in density estimates. Reasons for regional variation in densities relate to climate, vegetation group and topographical variation, as well as other variables, such as land use (Carver et al., 2021; Driessen, Dewar, Carver, Lawrence, & Gales, 2022). For example, Carver et al. (2021) found that temperature and rainfall are strongly linked to wombat counts. Furthermore, wombats prefer regions where they can both burrow and graze (Triggs, 2009), thus, mountainous terrain with topographical roughness is a less suitable habitat for them. However, evaluating these effects is beyond the scope of this study. Therefore, future research investigating climatic and landscape factors in relation to density at a regional scale could be of value.

Researchers and wildlife managers in Tasmania are commonly asked by the public and media how many wombats are in the state (Carver, personal communication, November 2021). Prior to this study, an estimate of population size was unknown. Considering the aforementioned likely overrepresentation and underrepresentation of particular habitat types surveyed, and uncertainties in wombat densities estimated among habitat types, there is an understandable level of uncertainty in wombat abundances estimated in this study. It is possible that our estimate of 840,665 (95% CI = 531,104–1,201,547) wombats on the Tasmanian mainland may be positively biased, and we considered more conservative wombat abundance estimates in the results for this reason. However, we also acknowledge that there is heterogeneity in when bare‐nosed wombats emerge from their burrows and how long they forage for each night, or even if they emerge from their burrows each night (Borchard et al., 2012; Simpson et al., 2016; Triggs, 2009). Such heterogeneity would reduce detection probability on spotlight surveys and negatively bias abundance estimates. Thus, all factors considered we think the credible intervals around our average estimate are likely either a conservative or a fair representation of the ‘true’ number of wombats on the Tasmanian mainland. Future research quantifying the relative proportion of wombats above ground over their circadian cycles would be valuable to enable a temporal correction factor to be applied to the statistical models, enhancing accuracy and confidence in estimating ‘true’ abundance.

Even though we estimated abundance for *V. u. tasmaniensis* across its entire geographical range, this was not the case for *V. u. ursinus* which also occurs on Maria Island in the south‐east of the state. Historically, *V. u. ursinus* occupied a wider geographical range, including other Bass Strait islands but were extirpated following European colonialism (Rounsevell et al., 1991). Twenty‐one wombats were translocated from Flinders Island to Maria Island in the early 1970s (Ingram & Kirkpatrick, 2013; Rounsevell, 1989). Ingram (2019) estimated the wombat population on Maria Island to be 2599 (95% CI = 2254–2858) in 2019, showing an increase since surveys began in 2010. Collectively, this suggests that the estimated population size for the *V. u. ursinus* subspecies on both Maria Island and Flinders Island is in the order of 75,000 individuals.

In a government report, Heard and Ramsey (2020) present the only other attempt to estimate abundance of a bare‐nosed wombat subspecies (*V. u. hirsutus*) over a large spatial scale (Victoria, Australia). Their valuable study modelled wombat abundance, using camera trapping data and distance modelling techniques, spanning a total time frame of 15 years, and derived estimates of wombat abundance across the state. Their state‐wide wombat abundance estimation was 432,595, with a 95% CI of 405,559–461,388. Victoria comprises more than three times the land area of Tasmania, and has had a history of significant human‐wombat conflict (Temby, 1998), which likely contributes to the lower overall wombat population estimate relative to Tasmania. There are also differences between the studies that we feel are important to acknowledge. Heard and Ramsey (2020) used camera traps for their study, and research suggests wombat detections can differ between camera trap and spotlight surveys (Driessen, Dewar, Carver, & Gales, 2022). Heard and Ramsey (2020) used a large number of wombat observations (7883) with camera trap placement biased towards the east and south‐east of Victoria (where the authors also estimated highest wombat abundance to occur) and our study was negatively biased towards the south‐west of Tasmania owing to a lack of roads in that part of the state (though wombats are known to occur there). Heard and Ramsey (2020) acknowledge their sampled data were largely restricted to forested public land and included few sites that were in farmland or heavily cleared areas, cautioning that abundance predictions across agricultural land should be treated with care. Indeed, the density estimate (0.28 ha^−1^) in Heard and Ramsey (2020) is slightly lower than some other smaller scale density estimates from within the state (Banks et al., 2002; Skerratt et al., 2004), suggesting the state‐wide bare‐nosed wombat abundance estimate may be an underestimate, much like our Tasmanian estimates could be underestimates. Nevertheless, our estimates for *V. u. ursinus* and *V. u. tasmaniensis* and the estimate from Heard and Ramsey (2020) for *V. u. hirsutus* in Victoria are highly valuable pieces of information. Further research to estimate abundance across the range of *V. u. hirsutus* would be valuable, particularly for New South Wales, the Australian Capital Territory and eastern South Australia, where the rest of the subspecies' range occurs.

Interestingly, we found the shape of the detection function differed between Flinders Island and mainland Tasmania (Figure 3), necessitating separate analyses. In addition, we found that the detectability changed over time for surveys conducted on Flinders Island; specifically, animals were more readily detected at further distances in later years. It is well known that miss‐specification of the detection function can lead to biased population estimates when using distance sampling methods (Buckland et al., 2001; Schmidt et al., 2012). Detection probability is a function of many factors (Thomas et al., 2002), such as observer skill, environmental variables (e.g. wind speed, temperature) and vegetation. Observer skills and environmental conditions on survey nights probably did not have a significant impact on counts as surveyors are trained and experienced, surveys were conducted at the same time of year, survey conditions were standardised to avoid weather likely to affect counts (e.g. rain and high winds) (Driessen & Hocking, 1992) and wombats are relatively large conspicuous animals. Furthermore, detection is not only facilitated by their size but also by their behaviour, that is wombats are generally tolerant of spotlights (Taylor, 1993), and exhibit little avoidance behaviour towards roads and vehicles (Burgin & Brainwood, 2008; Roger et al., 2007). Land‐use changes (e.g. land clearing for pasture) might have influenced the temporal shift on Flinders Island. Given we detected significant regional differences in detection, despite similar observers and methodology, we advocate that distance studies pay particular attention to this possible source of bias‐inducing variation.

## AUTHOR CONTRIBUTIONS

**Wiebke Knoblauch:** Conceptualization (lead); formal analysis (lead); methodology (lead); writing – original draft (lead); writing – review and editing (equal). **Scott Carver:** Conceptualization (lead); formal analysis (supporting); writing – review and editing (equal). **Michael M. Driessen:** Data curation (lead); writing – review and editing (equal). **Rosemary Gales:** Data curation (lead); writing – review and editing (equal). **Shane A. Richards:** Formal analysis (supporting); writing – review and editing (equal).

## CONFLICT OF INTEREST STATEMENT

The authors declare no conflict of interest.

## Data Availability

The data that support the findings of this study are available in Dryad at https://doi.org/10.5061/dryad.q83bk3jnz.

## APPENDIX A

**TABLE A1:** Area of broad vegetation groups on Flinders Island as defined by Kitchener and Harris (2013) and used in the TASVEG 4.0 dataset (Department of Natural Resources and Environment, 2020).

Vegetation group	Area (ha)	Area (%)
Dry eucalypt forest and woodland	28,815	21.2
Modified land	47,413	34.9
Moorland, sedgeland and rushland	1	0.0
Native grassland	1630	1.2
Non‐eucalypt forest and woodland	5459	4.0
Other natural environments	5121	3.8
Rainforest & related scrub	93	0.1
Salt marsh & wetland	4615	3.4
Scrub, heathland and coastal complexes	37,377	27.5
Wet eucalypt forest and woodland	5373	3.9
Total	135,897	100

**TABLE A2:** Area of broad vegetation groups on the Tasmanian mainland as defined by Kitchener and Harris (2013) and used in the TASVEG 4.0 dataset (Department of Natural Resources and Environment, 2020).

Vegetation group	Area (ha)	Area (%)
Dry eucalypt forest and woodland	1,515,063	23.1
Highland and treeless vegetation	135,549	2.1
Modified land	1,510,490	23.0
Moorland, sedgeland and rushland	596,629	9.1
Native grassland	121,130	1.9
Non‐eucalypt forest and woodland	179,259	2.7
Other natural environments	239,951	3.7
Rainforest and related scrub	757,028	11.5
Salt marsh and wetland	28,600	0.4
Scrub, heathland and coastal complexes	363,137	5.5
Wet eucalypt forest and woodland	1,114,717	17.0
Total	6,561,553	100

**TABLE A3:** Median densities (individuals/ha) of *Vombatus ursinus ursinus* on Flinders Island, including 95% CI.

Year	Median density (individuals/ha)	95% CI
2002	0.21	0.12–0.41
2003	0.31	0.19–0.62
2004	0.41	0.27–0.76
2005	0.54	0.33–1.09
2006	0.53	0.33–1.01
2007	0.37	0.22–0.72
2008	0.24	0.14–0.43
2009	0.27	0.16–0.47
2010	0.28	0.16–0.50
2011	0.21	0.12–0.41
2012	0.52	0.32–0.98
2013	0.55	0.34–1.08
2014	0.35	0.20–0.63
2015	0.40	0.22–0.74
2016	0.35	0.21–0.67
2017	0.78	0.49–1.41
2018	0.63	0.39–1.17
2019	0.46	0.27–0.94
2020	0.53	0.32–1.01

Abbreviation: 95% CI, 95% credible intervals.

**TABLE A4:** Median densities (individuals/ha) of *Vombatus ursinus tasmaniensis* on the Tasmanian mainland, including 95% CI.

Year	Median density (individuals/ha)	95% CI
2002	0.11	0.07–0.19
2003	0.11	0.06–0.17
2004	0.07	0.04–0.12
2005	0.10	0.06–0.17
2006	0.14	0.08–0.24
2007	0.10	0.06–0.16
2008	0.13	0.08–0.22
2009	0.12	0.07–0.21
2010	0.09	0.06–0.16
2011	0.09	0.06–0.17
2012	0.11	0.06–0.19
2013	0.11	0.07–0.19
2014	0.10	0.06–0.17
2015	0.14	0.08–0.24
2016	0.13	0.07–0.22
2017	0.13	0.08–0.23
2018	0.12	0.07–0.20
2019	0.14	0.09–0.24
2020	0.10	0.06–0.15

Abbreviation: 95% CI, 95% credible intervals.
